# Pseudoaneurysms of the lingual artery: two case reports and a systematic review

**DOI:** 10.1007/s00234-026-03932-x

**Published:** 2026-03-06

**Authors:** Liliana Igreja, Sara Costa, João Pedro Filipe, José Pedro Rocha Pereira, Francesco Diana

**Affiliations:** 1 Department of Neuroradiology, Hospital de Santo António, Porto, Portugal; 2 Department of Otorhinolaryngology, Hospital de Santo António, Porto, Portugal; 3 Interventional Neuroradiology, Vall d’Hebron Hospital Universitari, Barcelona, Spain

**Keywords:** Pseudoaneurysm, Lingual artery, Oropharyngeal haemorrhage, Parent vessel occlusion, Endovascular treatment.

## Abstract

**Background:**

Pseudoaneurysms of the lingual artery (PLAs) are rare vascular lesions that may develop secondary to trauma, infection, or head and neck surgical and oncologic procedures. Although uncommon, they constitute a potentially life-threatening cause of oropharyngeal hemorrhage. Their heterogeneous etiologies and variable presentations contribute to the absence of standardized management guidelines.

**Purpose:**

To provide a comprehensive analysis of the diagnosis and treatment of published PLAs, alongside the description of two additional clinical cases.

**Data sources:**

A systematic review of the literature was conducted according to PRISMA guidelines and using the PubMed and Scopus databases.

**Study selection:**

Were included 45 published cases comprising 55 patients with PLAs, plus two novel unreported cases.

**Data analysis:**

Data regarding patient demographics, etiology, clinical presentation, imaging, aneurysm location, treatment strategy, and outcomes were extracted and analysed.

**Data synthesis:**

The mean patient age was 37.6 years, showing a bimodal distribution: younger patients commonly presented with post-tonsillectomy or traumatic pseudoaneurysms, while older individuals were more often affected after malignancy or oncologic surgery. Haemorrhage was the primary presentation (75.4%) and 38.6% of all cases followed tonsillectomy. Four cases were asymptomatic. Most lesions arose from the main trunk of the lingual artery (42.1%) or unspecified segments of the vessel (36.8%). Endovascular treatment (EVT) was successful in 77.2% of cases, in which parent vessel occlusion (PVO) proved to be the most reliable and effective technique (88.6%). Ultrasound-guided percutaneous thrombin injection was effective in two patients. EVT failed in two cases due to catheterization difficulties and arterial dissection. Mortality was low (3.5%).

**Limitations:**

Most data derive from heterogeneous case reports and series with limited sample sizes and variable reporting standards.

**Conclusions:**

PLAs are rare but potentially life-threatening causes of oropharyngeal bleeding. Prompt diagnosis and EVT, particularly PVO, are essential for successful management. Extensive collateral circulation supports safe vessel exclusion. Multidisciplinary coordination optimizes patient outcomes.

**Supplementary Information:**

The online version contains supplementary material available at 10.1007/s00234-026-03932-x.

## Introduction

Aneurysms involving branches of the external carotid artery (ECA) account for less than 1% of all arterial aneurysms [1–4]. Among these, pseudoaneurysms of the lingual artery (PLA) are exceedingly rare. The distinction between a true aneurysm and a pseudoaneurysm lies in the integrity of the vessel wall layers. In pseudoaneurysms, there is a post-traumatic or post-infectious disruption of the elastic laminae, tunica media, and intima, whereas true aneurysms retain all arterial wall layers, although often attenuated.

The lingual artery is typically a branch of the ECA, arising between the superior thyroid and facial arteries, although anatomical variants are common. In some individuals, it may arise from a common trunk with the facial artery, known as the linguofacial trunk. Additionally, it can originate together with the superior thyroid and facial arteries from a single common trunk, referred to as the thyrolinguofacial trunk [5].

The artery courses through three segments: mandibular, lingual, and sublingual, supplying the tongue, floor of the mouth, and adjacent structures. Awareness of these segments and their potential anastomotic connections with the contralateral lingual and facial arteries is crucial for planning endovascular interventions and understanding collateral circulation.

Most PLAs are iatrogenic in origin, typically following procedures such as tonsillectomy or radiofrequency ablation, but they may also arise secondary to direct tumour invasion, infection, or, less frequently, blunt or penetrating trauma [6–13]. Clinically, PLAs can present with severe haemorrhage into the oral cavity or oropharynx, sublingual haematoma leading to upper airway obstruction (the so called pseudo-Ludwig phenomenon [14–16]), or submental swelling, depending on their location. In some cases, they may remain asymptomatic. Massive oropharyngeal bleeding constitutes a life-threatening emergency associated with high morbidity and mortality. Because of their rarity, evidence regarding the optimal management of PLAs, particularly using endovascular treatment (EVT), remains limited, with a small number of case reports published to date.

In this paper, we report two additional rare cases of PLAs, one secondary to recurrent laryngeal carcinoma and the other post-procedural, both successfully treated with EVT. Furthermore, we provide an updated review of the limited available literature on PLA management (Table 1), emphasizing the feasibility and safety of lingual artery sacrifice and discussing the compensatory role of regional collateral networks in maintaining perfusion.

**Table 1:** **Comprehensive overview of all published case reports documenting pseudoaneurysms of the lingual artery**. **The table details demographic data**,** underlying etiologies**,** clinical manifestations**,** imaging modalities used for diagnosis**,** vascular localization**,** therapeutic interventions and reported clinical outcomes**. *Abbreviations: CE-CT: contrast-enhanced computed tomography; CTA: computed tomography angiography; DSA: digital Subtraction angiography; ECA: external carotid artery; MR: magnetic resonance; MRA: magnetic resonance angiography; NA: not available; OSAS= obstructive sleep apnea syndrome; TCRF= temperature-controlled radiofrequency reduction; TORS= transoral robotic surgery; US: ultrasound*

	STUDY	STUDY TYPE	AGE	**SEX**	**CLINICAL MANIFESTATION**	**DIAGNOSIS**	**ETIOLOGY**	**PSEUDOANEURYSM LOCALIZATION**	**TREATMENT**	**OUTCOME**
1	Gomori JM et al. 1983 [17]	Case report	38	M	1-year long right submandibular pulsatile mass, with shortness of breath	CTA, DSA	Idiopathic	Proximal right lingual artery	Unspecified surgical procedure	Unreported follow-up
2	Orron DE et al. 1988 [18]	Case report	71	F	Large pulsatile left submandibular mass, with acute expansion and dyspnea	US, CE-CT, DSA	Idiopathic	Left lingual artery	Surgical proximal ligation	Unreported follow-up
3	Maurer et al. 1989 [19]	Case report	18	M	Sudden oral bleeding after tonsillectomy	DSA	Iatrogenic – Post-tonsillectomy	Main trunk of the left lingual artery	Coiling of the parent artery	Unreported follow-up
4	Schroth G et al. 1991 [9]	Case report	42	M	Massive oral bleeding 1 month after chemoradiotherapy for a base of tongue carcinoma	Endoscopy, DSA	Neoplasm-associated/Iatrogenic – Post-Chemoradiotherapy	Bifurcation of deep and dorsal branches of the left lingual artery	Coiling of the parent vessel	No further hemorrhagic complications
5	Adib A et al. 1993 [20]	Case report	76	F	Double episode of ecchymosis of the neck and oral cavity in patient without comorbidities	CE-CT, DSA	Bilateral idiopathic	Bilateral sublingual arteries	Unsuccessful coiling of the right one, followed by surgical proximal ligation	No recurrence within 6 months follow-up. Patient refused further treatment
6	Salgarelli A et al. 1997 [21]	Case report	27	M	Patient surgically treated for massive abscess involving the left base of tongue. Two weeks US follow-up showed the pseudoaneurysm	Echo-Color-Doppler, DSA	Post-infectious/Iatrogenic	Dorsal artery of the tongue	Gelfoam particles	15 days US follow-up showed the occlusion of the pseudoaneurysm and the patency of the arterial trunk
7	Mitchell RB et al. 1997 [22]	Case report	3	F	Massive oral bleeding during an adenotonsillectomy	DSA	Iatrogenic – Post-tonsillectomy	Right main trunk of the lingual artery	Coiling of the parent vessel	Uncomplicated post-operative period
8	Amirjamshidi A et al. 2000 [23]	Case report	17	NA	Post-traumatic pulsatile mass	DSA	Post-traumatic	Lingual artery	Surgical excision	Unreported follow-up
9	Simoni P et al. 2001 [24]	Case report	8	F	Sudden oral bleeding after tonsillectomy	DSA	Iatrogenic – Post-tonsillectomy	Main trunk of the right lingual artery	Coiling of the parent artery	Uncomplicated post-operative period. Unreported follow-up
10	Roelke et al. 2004 [25]	Case report	21	F	Oral bleeding and submandibular swelling 6 days after tonsillectomy	Echo-Color-Doppler, DSA	Iatrogenic - Post-tonsillectomy	Main trunk of the left lingual artery	Gelfoam particles and coiling of the parent artery	Neither recurrence nor deficits reported at 5 years follow-up.
11	Walshe P et al. 2005 [7]	Case report	30	M	Massive oral bleeding 10 days after uvulopalatoplasty and tonsillectomy for OSAS	CTA, DSA	Iatrogenic – Post-tonsillectomy	Muscular branch of the left lingual artery	Unspecified embolization	Uncomplicated post-operative period and no bleeding recurrence at 2 weeks follow-up
12	Herzog M et al. 2006 [26]	Case report	34	M	Military-associated penetrating cervical trauma	DSA	Iatrogenic – post-TCRF	Main trunk of the right lingual artery	Coiling of the parent artery	Neither recurrence nor deficits reported after 4 weeks.
13	Charles JF et al. 2006 [12]	Case (**1**) selected from a case series	NA	NA	Military-associated penetrating cervical trauma	CTA, DSA	Post-traumatic	Right lingual artery	Coiling of the parent vessel	Unreported follow-up
14	Li SH et al. 2006 [27]	Case report	61	M	Right hypopharyngeal bulging mass after chemoradiotherapy for nasopharyngeal carcinoma	Nasopharyngoscopy, CTA, DSA	Neoplasm-associated/Iatrogenic – Post-Chemoradiotherapy	Right linguofacial trunk	Coiling of the parent vessel	Complete resolution of the hypopharyngeal bulging mass at 3 months follow-up
15	Van Cruijsen N et al. 2007 [28]	Case report	3	M	Sudden and massive oral bleeding 9 days after tonsillectomy	DSA	Iatrogenic – Post-tonsillectomy	Main trunk of the right lingual artery	Coiling of the parent artery	Unreported follow-up
16	Matsumoto T et al. 2007 [29]	Case report	69	F	Sudden major haemoptysis 5 weeks after pharyngolaryngoesophagectomy for esophageal squamous cell carcinoma	DSA	Iatrogenic – Post-pharyngolaryngoesophagectomy	Main trunk of the left lingual artery	Glue	Neither recurrence nor deficits reported at 2.5 years follow-up.
17	Handa KK et al. 2008 [30]	Case report	20	M	Submental swelling and mass effect on the tongue after penetrating trauma of the head and neck	CE-CT	Post-traumatic	Main trunk of the left lingual artery	Surgical ligation of lingual artery	Unreported follow-up
18	Windfuhr JP et al. 2010 [31]	Case report and review of post-tonsillectomy pseudoaneurysms	5	F	Sudden oral bleeding after tonsillectomy	DSA	Iatrogenic – Post-tonsillectomy	Main trunk of the lingual artery	Coiling of the parent artery	Unreported follow-up
19	Brindle et al. 2010 [32]	Case report	65	F	Presurgical evaluation with CE-CT scan of the neck in patient with hyperparathyroidism. Incidental finding	CE-CT	Idiopathic	Left sublingual artery	Aneurysmal coiling	Unreported follow-up
20	Murono S et al. 2011 [33]	Case report	64	M	Sudden oral bleeding 10 weeks after intra-arterial chemotherapy for squamous cell carcinoma of the left side of the tongue	Fyberoptic laryngoscopy, DSA	Iatrogenic – Post-Chemoembolization	Main trunk of the left lingual artery	Coiling of the parent artery	Neither recurrence nor deficits reported at 7 months follow-up
21	Kaschner M et al. 2011 [34]	Case report	22	M	Submental swelling after road trauma	CE-CT	Post-traumatic	Main trunk of the right lingual artery	Guided ultrasound percutaneous thrombin injection	Reduction of the aneurysmal dimensions after 4 weeks
22	Atmaca S et al. 2011 [35]	Case report	3.5	F	Severe oral bleeding 6 days after bilateral tonsillectomy	CTA, DSA	Iatrogenic – Post-tonsillectomy	Right linguofacial trunk	Coiling of the parent artery	No bleeding recurrence at 1-month follow-up
23	Fassnacht W et al. 2012 [36]	Case report and review	23	F	Sudden onset oral bleeding 4 days after tonsillectomy	DSA	Iatrogenic – Post-tonsillectomy	Main trunk of the left lingual artery	Aneurysmal coiling + ECA retrograde coiling	Odynophagia and dysphagia 11 months after embolization due to coils extrusion
24	Badloo K et al. 2012 [37]	Case report	20 months	F	Submandibular swelling and fever in patient with mycotic pseudoaneurysm	CTA	Post-infectious (mycotic)	Main trunk of the right lingual artery	Surgical ligation ECA	Unreported follow-up
25	Masella PC et al. 2014 [38]	Case report	26	M	Oropharyngeal swelling after penetrating trauma of the head and neck	Echo-Color-Doppler	Post-traumatic	Main trunk of the right lingual artery	Guided ultrasound percutaneous thrombin injection	Unreported follow-up
26	Manzato L et al. 2015 [39]	Case report	19	F	Oral bleeding 20 days after left tonsillectomy	DSA	Iatrogenic – Post-tonsillectomy	Left linguofacial trunk	Coiling of the parent artery	Uncomplicated post-operative period
27	Iwai T et al. 2016 [40]	Case report	80	M	Incidental finding during evaluation for tongue cancer. No previous history of trauma, infection, or surgery	CTA, DSA	Bilateral Idiopathic	Bilateral lingual arteries	Conservative	Partial glossectomy for tongue cancer, with an uneventful post-operative period. Regular radiological follow-up on the small unruptured aneurysms was performed
28	Margallo L et al. 2017 [41]	Case report	45	M	Life-threatening hemorrhage 14 days after surgical repair of facial trauma	CTA, DSA	Post-traumatic	Right lingual artery	Coiling of the parent artery	Uncomplicated post-operative period
29	Cheng L et al. 2017 [10]	Case series (**2**)	53	M	Oral bleeding after chemoradiotherapy for an inoperable carcinoma of the posterior tongue	US, CT, DSA	Neoplasm-associated/Iatrogenic	Right lingual artery	Glue	No bleeding recurrence reported
			73	M						
								Unspecified lingual artery	Glue and coiling of both lingual arteries and sublingual anastomoses from both facial arteries	
30	Hassan F et al. 2019 [42]	Case series (**10**)	39	M	Oral bleeding after surgery of carcinoma of the mandibular alveolus	DSA	Iatrogenic – Post-tonsillectomy	Proximal lingual arteries (70%) | Common linguofacial trunks (30%)	80% Glue | 20% coiling of the parent vessel	No bleeding recurrence reported
31	Ramírez-Ramírez MM et al. 2019 [43]	Case report	39	M	Delayed post-tonsillectomy bleeding	Endoscopy, CTA, DSA	Idiopathic	Right lingual artery	Failed embolization, followed by surgical ligation of the tirolingual trunk	Uncomplicated post-operative period
32	Leedman SR et al. 2019 [44]	Case report	61	F	Melena and hematemesis, followed by massive oral bleeding	CTA, DSA	Iatrogenic – Post-TORS/Neoplasm-associated	Left lingual artery	Local suture and unsuccessful embolization due to vessel dissection during catheterization (which stopped the active bleeding	Recurrent bleeding at day 3, which resolved spontaneously
33	Espallargas I et al. 2020 [45]	Case report	89	F	Massive haematemesis 3 days after TORS for a in situ carcinoma, likely exacerbated by Fibromuscular Dysplasia	CTA	Neoplasm-associated	Lingual artery	Conservative(comorbidities)	Death from hemorrhage
34	Rathod R et al. 2021 [46]	Case report	31	M	Oral bleeding in oropharyngeal carcinoma	CTA, DSA	Post-traumatic	Lingual artery	Gelfoam particles and tongue laceration repair	No bleeding recurrence at 1-month follow-up
35	Ali I et al. 2022 [47]	Case report	42	M	Massive oral bleeding after facial trauma, with tongue laceration	CTA, DSA	Neoplasm-associated	Lingual artery	Unspecified embolization	Unreported follow-up
36	Yamamoto N et al.2022 [48]	Case report	55	F	Oral bleeding associated with squamous cell carcinoma of tongue on chemoradiotherapy	CTA	Idiopathic	Lingual artery	Embolization failed. Surgical excision	Unknown
37	Singh SP et al. 2022 [49]	Case report	1	M	Weeks of gradually progressive right submandibular swelling after blunt trauma	CTA, DSA	Post-traumatic	Right lingual artery	Coiling of the parent vessel	Unreported follow-up
38	Maleux O et al. 2022 [50]	Case report	68	M	Hematemesis 12 days after laser resection of an erythroplakia area on the right site of base of tongue. Previous history of local squamous cell carcinoma treated with surgery and radiotherapy	CTA, DSA	Neoplasm-associated/Iatrogenic – Post-surgery and/or radiotherapy	Right lingual artery	Glue	Focal and superficial ulceration at the level of the embolized bleeding point, with local pain, managed conservatively
39	Bravo J et al. 2022 [51]	Case report	NA	NA	Oral bleeding	US	Idiopathic	Left lingual artery	Glue - via direct percutaneous access (US-guided)	Uncomplicated post-procedure period
40	Ahmed F et al. 2023 [52]	Case report	61	F	Left-sided tongue pain and swelling one week after a tooth extraction	CTA, MR/MRA, DSA	Iatrogenic –Post tooth extraction	Left lingual artery	Onyx 18	Complete pseudoaneurysm occlusion at 1-month follow-up
41	Trinidad JS et al. 2024 [53]	Case report	25	M	NK-T cell tongue lymphoma presented with tongue pain and bleeding, progressing to haemorrhagic shock	CTA, DSA	Neoplasm-associated	Right lingual artery	Unspecified embolization	Uncomplicated post-procedure period
42	Daou et al. 2024 [54]	Case report	19	F	Spontaneous oral cavity bleeding of 5 days duration, after a self-resolving viral tonsillitis	Flexible fiberoptic laryngoscopy, CTA, DSA	Post-infectious	Tonsillar branch of the right lingual artery	Coiling of the parent vessel	Uncomplicated post-procedure period and no bleeding recurrence at
43	Buxo Z et al. 2025 [55]	Case report	20	M	Major oral bleeding 15 days after surgical repair of multiple gunshot facial wounds	CTA, DSA	Post-traumatic/Iatrogenic	Lingual artery	Coiling of the parent artery	Unknown
44	Yang X et al. 2025 [56]	Case (**1**) selected from a case series	42	M	3 episodes of oral bleeding 6 days post-tonsillectomy (for tonsillar tumor)	CTA, DSA	Iatrogenic – Post-tonsillectomy	Lingual artery	Glue	No recurrence of bleeding or adverse events
45	Bhali HE et al. 2025 [57]	Case report	20	M	Cervicofacial cellulitis, followed by profuse haematemesis and haemorrhagic shock	CTA, DSA	Post-infectious	Right lingual artery	Coiling of the parent artery	Disseminated intravascular coagulation and death following refractory shock
46	Our Case 1	Case report	70	M	Sudden oral bleeding two months after total laryngectomy in a patient with epidermoid carcinoma of the larynx	CTA, DSA	Iatrogenic – Post-pharyngolaryngoesophagectomy	Main trunk of the right lingual artery	Glue	No recurrence at 2 months follow-up
47	Our Case 2	Case report	64	M	Sudden massive oral bleeding associated with recurrent squamous cell carcinoma of larynx	CTA, DSA	Neoplasm-associated/Iatrogenic – Post surgery	Main trunk of the right lingual artery	Glue	No recurrence at 6 months follow-up

## Materials and methods

A literature review on the management of PLAs was conducted through systematic searches of the PubMed and Scopus databases. The search strategy included the keywords “lingual artery pseudoaneurysm(s)”, “lingual artery aneurysm(s)” and “lingual artery embolization”. The review process followed the ”Preferred Reporting Items for Systematic Reviews and Meta-Analyses” (PRISMA) guidelines [58], including the stages of publication search, eligibility assessment, data collection, extraction, analysis, and synthesis of the final systematic review report.

All studies describing non-surgical treatments for lingual artery aneurysms or pseudoaneurysms identified through the predefined search strategy were considered eligible (*n* = 37). Additionally, articles describing surgical management of PLAs, identified through the same keyword-based search but not specifically sought as a separate category, were included in the discussion for comparative purposes (*n* = 8). Articles published in languages other than English or lacking an abstract or full text were excluded (*n* = 8).

## Results

A total of 45 previously published articles, comprising 55 patients treated with either minimally invasive techniques (endovascular or percutaneous) or surgical excision, together with the two new clinical cases from this study, were included in the quantitative analysis and are summarized in Table 1. The mean age of the patients was 37.6 ± 25.2 years (range: 1–89 years), after excluding two patients with unreported age. Among the 57 patients, 34 (59.6%) were male, and three had no sex information available.

### Etiology and diagnosis

Among the 57 reported PLAs, iatrogenic causes were frequent (*n* = 28; 49.1%), predominantly following tonsillectomy or related oropharyngeal procedures (*n* = 22; 38.6%) [7, 9, 22, 24, 25, 28, 31, 35, 36, 39, 42, 56]. Another six patients (10.5%) had lesions secondary to other therapeutic interventions, including transoral robotic surgery [44], laryngectomy [29], intra-arterial chemoembolization [26], transcutaneous radiofrequency [33], and tooth extraction [52]. A distinct subgroup of seven patients (12.3%) presented pseudoaneurysms with a possible dual etiology [19, 27, 50, 56, 59], being both neoplasm-associated and iatrogenic, typically following chemoradiotherapy or oncological surgery. Purely neoplasm-associated pseudoaneurysms were identified in three patients (5.3%) [45, 47, 53], probably due to tumour-related invasion. Trauma accounted for eight cases (14%) [12, 23, 30, 34, 38, 41, 46, 49] ,including both penetrating and blunt craniofacial trauma, while one case (1.8%) exhibited a possible mixed post-traumatic and iatrogenic origin [55]. Idiopathic pseudoaneurysms occurred in seven patients (12.3%) [17, 18, 20, 32, 40, 48, 51] , including rare bilateral lesions [20, 40] and one associated with Fibromuscular Dysplasia (FMD) [48]. Infectious causes were identified in three patients (5.3%) [21, 37, 57], with one PLA considered to be of mycotic origin [37] and other whom was categorized as post-infectious/iatrogenic after surgical drainage of a large abscess [21].

Diagnosis relied heavily on digital subtraction angiography (DSA), being performed in 49 patients (86%), with contrast-enhanced computed tomography (CE-CT)/computed tomography angiography (CTA) used in 30 patients (52.6%) often as the first-line investigation. Ultrasound (US) or echo-colour-doppler was described in 7 patients (12.3%), mainly for initial assessment of submandibular or sublingual swellings. Endoscopic visualization was mentioned in 5 patients (8.8%) for direct localization of the bleeding source. Finally, magnetic resonance (MR)/magnetic resonance angiography (MRA) was used only once (1.8%) as a complementary diagnostic tool [52].

### Localization and clinical presentation

PLAs most frequently originated from the main trunk / proximal lingual artery (*n* = 24; 42.1%), followed by “lingual arteries” not further specified (*n* = 21; 36.8%), a common linguofacial trunk (*n* = 6; 10.5%), sublingual branches (*n* = 2; 3.5%), dorsal branch / bifurcation of dorsal and deep branches (*n* = 2; 3.5%), muscular branches (*n* = 1; 1.7%) and a tonsillar branch (*n* = 1; 1.7%).

Oral or oropharyngeal haemorrhage was the most common manifestation, reported in 43 patients (75.4%), frequently following tonsillectomy. Within this group, a subset of 11 patients (19.3%) reported “massive” haemorrhage or shock [7, 9, 22, 28, 41, 43, 44, 46, 53, 57], representing life-threatening situations that required emergent intervention. Pulsatile masses or swelling, located in the submandibular, sublingual, submental or cervical regions, were observed in 10 patients (17.5%) [18, 21, 27, 30, 34, 37, 38, 49, 52] . Incidental and asymptomatic PLAs were reported in 3 patients (5.3%) [32,40, 48]. One traumatic case [12] could not be classified due to an inconclusive clinical presentation.

### Treatment

A successful endovascular treatment (EVT) of PLAs was performed in 44 patients (77.2%), predominantly through parent vessel occlusion (PVO) in 39/44 cases (88.6%) that was achieved by coils in 19/39 cases (48.7%), n-butyl-2-cyanoacrylate (NBCA) glue in 15/39 cases (38.5%), gelfoam particles of unspecified size in 2/39 cases (5.13%) and Onyx™ 18 (*Medtronic*) in 1/39 case (2.56%). In isolated cases a combined technique was applied, including gelfoam particles and coiling [25], and glue and coiling [59]. Selective aneurysmal coiling was performed in 2/44 cases (4.55%) [32, 26]. Additionally, one case reported a successful direct percutaneous access followed by a glue injection [51]. Overall, embolization achieved immediate hemostasis in nearly all patients, failing in two cases due to catheterization failure and lingual artery dissection. One of these patients subsequently underwent surgical ligation [20], and in the other the bleeding ceased spontaneously, allowing for conservative management [44]. In two cases (3.51%) a guided US percutaneous of thrombin injection resulted in a successful treatment [34, 38]. Surgical ligation or excision of the affected lingual artery or the ECA was performed in 8 cases (14.04%) [17, 18, 20, 23, 30, 37, 43, 48], either as primary therapy or as a salvage procedure after failed embolization, infection or limited interventional resources. On the other hand, conservative management was uncommon, reported in two patients (3.51%) [40, 45] and related to a small incidental lesion and to patient’s comorbidities. Most patients experienced complete resolution of bleeding and no recurrence during follow-up, with a mean reported follow-up time of eight months (2 weeks − 5 years). Reported complications were rare, including recurrent bleeding [44], localized pain and superficial ulceration [50], or delayed coil extrusion after treatment [36]. Mortality was exceptional, limited to two cases (3.51%): one due to disseminated intravascular coagulation secondary to refractory shock [57], and another resulting from haemorrhagic shock in a pseudoaneurysm managed conservatively [45]. Additionally, one patient with bilateral idiopathic lesions refused the treatment of the second one [20].

## Discussion

### Population and etiology

The mean age of 37.6 years reflected a bimodal distribution consistent with previous reports, with younger patients typically developing PLAs after tonsillectomy, trauma, or infectious processes, whereas older individuals more commonly presented with PLAs associated with neoplastic disease or its treatment [13, 42]. Idiopathic pseudoaneurysms accounted for 12.3% of cases. Some reported pseudoaneurysms may represent “true” aneurysms, particularly those lacking a clear history of acute vessel wall disruption, though this distinction is often theoretical without histopathological confirmation. In the absence of identifiable precipitating factors, an underlying connective tissue disorder should also be considered as a potential cause. Conditions such as Marfan syndrome, Ehlers–Danlos syndrome or Osteogenesis imperfecta have been associated with increased vascular fragility and spontaneous arterial rupture [60], which could theoretically contribute to pseudoaneurysm formation.

### Clinical presentation and diagnosis

PLAs most often manifested through haemorrhage and 36.8% of all cases followed a tonsillectomy, although asymptomatic cases may occasionally occur. Post-tonsillectomy haemorrhage is reported in approximately 3–4% of procedures [13], with pseudoaneurysmal bleeding occurring immediately after surgery or days to years later [13, 42]. In our review, four patients bled during or immediately after surgery [19, 22, 24, 31], while the remain presented with delayed haemorrhage 3 to 20 days later. Asymptomatic PLAs were rare, one likely post-infectious or iatrogenic [21], whereas three were considered idiopathic and incidental [32, 40, 48].

DSA was the diagnostic modality of choice (86%), particularly in emergency contexts for massive bleeding or haemorrhagic shock. When the patient’s haemodynamic status allowed, CTA and Echo-Color-Doppler were valuable complementary tools for lesion identification and pre-therapeutic planning.

Interestingly, FMD was mentioned in two patients of this review [44, 48], supporting the hypothesis that structural or connective tissue abnormalities might play a contributory role in the pathogenesis of idiopathic PLAs.

### Treatment

Currently, there is no established standard of care for PLAs management. Therapeutic strategies were highly individualized and heterogeneous, depending on etiology, clinical presentation, anatomical accessibility, institutional expertise, and hemodynamic stability. Evidence from large series of extracranial carotid aneurysms [61] suggests EVT is preferred for iatrogenic and traumatic pseudoaneurysms, while open surgical repair is favoured for “true” aneurysms, particularly those related to connective tissue disorders, where the risk of vessel wall fragility or procedural failure may be higher [60, 62]. Nevertheless, this does not constitute an absolute contraindication for EVT [62], since advances in microcatheter technology, embolic agents, and flow-directed materials have significantly improved the safety profile and technical success.

Moreover, studies demonstrate that EVT can achieve durable exclusion and haemorrhagic control with lower morbidity than surgery, especially in complex post-surgical or irradiated fields. However, most available data come from heterogeneous series [61, 63] with limited representation of ECA branch lesions, reducing applicability to PLAs. Consequently, while general carotid aneurysm principles may guide decision-making, PLAs management requires individualized planning, balancing anatomical accessibility, collateral circulation, and safe PVO feasibility.

In our systematic review, a successful EVT of PLAs was performed in 77.2% and PVO emerged as the safest and most effective treatment approach in 88.6% of these cases. In three particular patients [7, 47, 53] the type of embolization approach was not specified, yet complete aneurysmal exclusion was documented. Selective coiling of the aneurysmal sac was performed in two patients [32, 36], both achieving immediate angiographic occlusion. Conversely, EVT failed in two cases due to catheterization difficulties and dissection of the lingual artery.

**Fig. 1:**
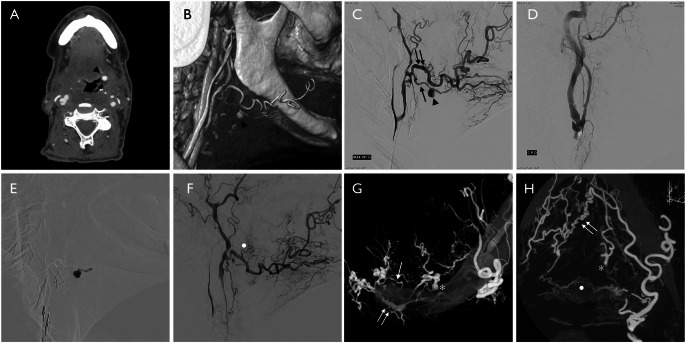
**(A)** Axial CTA, showing the left PLA close to the ipsilateral vallecula (*arrowhead*); **(B)** CTA 3D reconstruction showing the left PLA (*arrowhead*) in the medial portion of the suprahyoid region; **(C)** DSA, lateral view with injection of the left ECA showing the pseudoaneurysm (*arrowhead*) of the main trunk of the lingual artery (*arrow*) and the ipsilateral facial artery (*double arrow*); **(D)** DSA, lateral view with injection of the right ECA showing the absence of the ipsilateral facio-lingual trunk; **(E)** superselective injection of diluited Glue within the pseudosac and the distal portion of the parent artery; **(F)** DSA, lateral view with injection of the left ECA after embolization showing the complete occlusion of the PLA and the proximal part of the lingual artery; dorsal tongue arcade (*white dot*); **G-H)** XperCT frontal Gradient-shaded view (**G**) and axial MIP-reconstructions (**H**) showing the three arterial anastomotic arcades: (**i**) the submental arcade (*white double arrow*) between the two submental branches of the facial arteries, supplying the contralateral facial and lingual arteries; (**ii**) the sublingual arcade (*white arrow*) between the two sublingual branches of the lingual arteries supplying the left lingual artery and (*iii*) the dorsal tongue arcade (*white dot*) between the two dorsal branches of the lingual arteries. The asterisk points the distal segment of the left lingual artery

The safety and efficacy of PVO in the treatment of PLAs is largely attributable to the extensive ipsilateral and contralateral collateral networks between the lingual and facial arteries [5]. As shown in our **case report 1**, three major arterial arcades provided robust side-to-side anastomotic circulation: the dorsal lingual arcade (Fig. 1F, H), located along the dorsum of the tongue; the sublingual arcade, coursing through the sublingual space and derived from the lingual artery; and the submental arcade, arising from the facial artery (Fig. 1G, H). In addition, an anastomotic ring surrounding the sublingual gland connects branches of the lingual and facial arteries, further enhancing cross-flow compensation. These communications allow for rapid reversal of flow following vessel occlusion [5, 64], where after embolization the left facial artery alone maintained retrograde perfusion of the entire tongue and floor of the mouth.

Moreover, in two patients presenting with large aneurysmal dilatations (> 1.5 cm) and evident submandibular swelling, US-guided percutaneous thrombin injection was employed as an alternative technique [34, 38], resulting in favourable and durable outcomes. Finally, surgical ligation of the lingual artery and/or ECA was performed in eight patients, either as primary therapy or as a salvage procedure after failed embolization, infection or limited interventional resources, resulting in generally favourable outcomes, with haemostatic success and an uneventful recovery.

Overall, the available evidence suggests that EVT, particularly PVO, represents a feasible and effective therapeutic option for most PLAs, with selective catheterization being frequently achievable and without clinically significant ischemic complications. Nevertheless, the heterogeneity of reported cases and the limitations in the quality and completeness of available data preclude drawing definitive conclusions regarding the preferential role of endovascular treatment as a first-line strategy. Surgical management remains a valuable alternative in cases of technical failure, infection, unfavorable vascular anatomy, or when endovascular treatment is not feasible or contraindicated.

### Case report 1

A 70-year-old man with a history of laryngeal epidermoid carcinoma previously treated with chemoradiotherapy developed a radiation-induced laryngeal stenosis, requiring tracheotomy and gastrostomy. During follow-up, an endoscopic biopsy revealed extensive laryngeal chondronecrosis, prompting a total laryngectomy without complications. Shortly after discharge, he experienced two episodes of oral bleeding. Examination showed left retromandibular swelling, dyspnoea and tachycardia, though he remained hemodynamically stable (haemoglobin 10.1 g/dL). Intubation was performed through the tracheotomy, and fiberoptic endoscopy revealed no active bleeding. CTA identified a 6 mm pseudoaneurysm of the main trunk of the left lingual artery (Fig. 1A, B) and he was referred for a DSA and endovascular intervention. The procedure was performed under general anaesthesia via right transfemoral access. A vertebral 4 F catheter (*Terumo*) was used for the complete pre-treatment evaluation and the left ECA catheterization, which confirmed the pseudoaneurysm of the main trunk of the left lingual artery (Fig. 1C), the post-irradiation occlusion of the right linguofacial trunk (Fig. 1D) and the presence of three types of regional anastomoses: the sublingual and submental arcades and the arcade of the base of the tongue. A microcatheter Magic 1.2 F (*BALT*) was positioned into the aneurysmal sac using a Hybrid 0.007 guidewire (*BALT*), and 0.4 ml of diluted NBCA glue (*Glubran2*) was injected, selectively occluding the pseudoaneurysm and a distal short segment of the parent artery (Fig. 1E). Final DSA runs documented complete exclusion of the pseudoaneurysm with preserved distal lingual branches through the regional anastomoses (Fig. 1F-H). The patient was discharged without neurological deficits and no rebleeding occurred until the latest follow-up.

### Case report 2

**Fig. 2:**
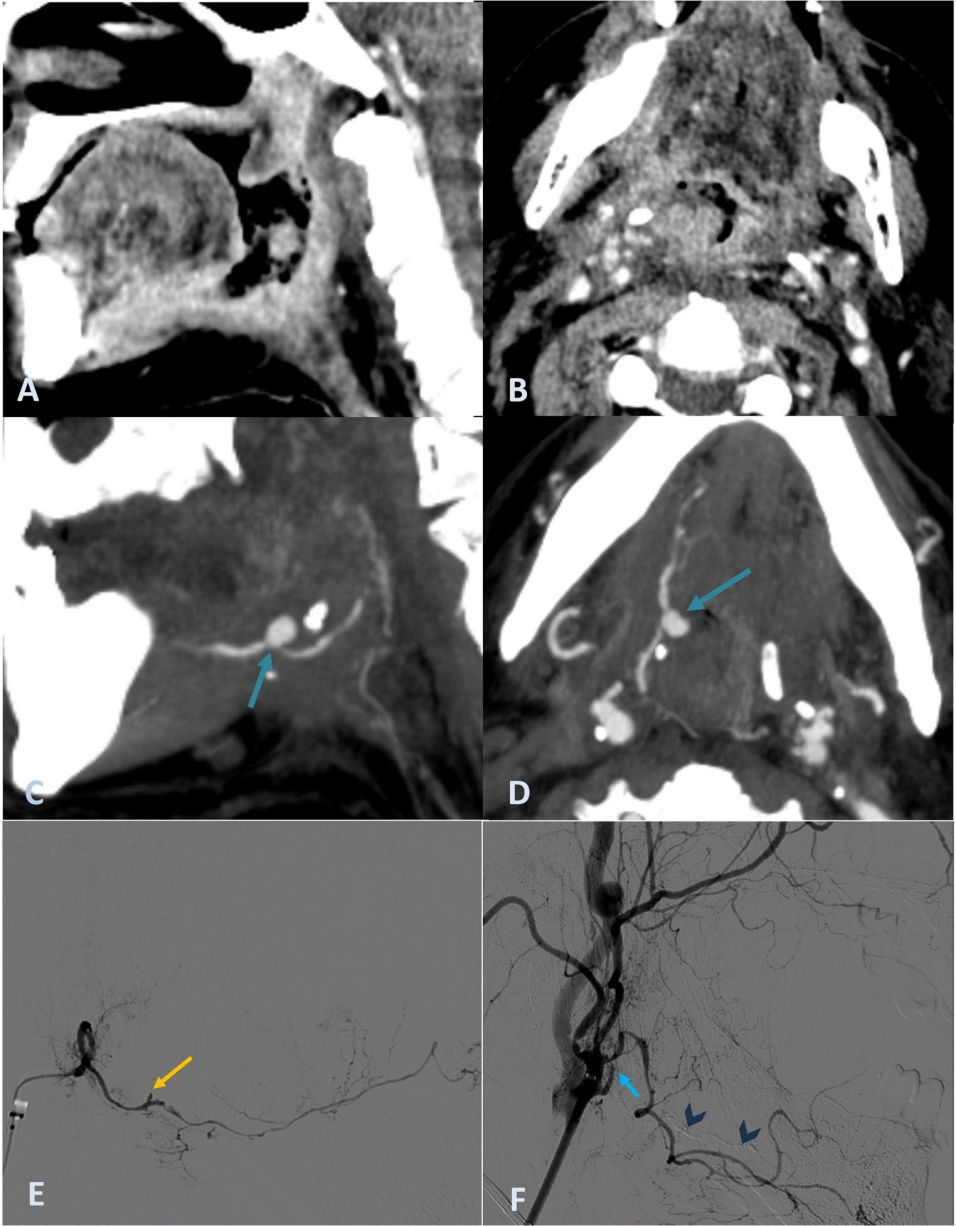
CE-CT images in sagittal (**A**) and axial (**B**) planes showing an infiltrative lesion involving the neopharynx walls, with extension into the right tonsillar region and tongue base, consistent with recurrent tumour. CTA in sagittal (**C**) and axial (**D**) planes demonstrating a pseudoaneurysm of the right lingual artery at the tongue base (*blue arrows*), located beneath the area of tumour ulceration. DSA identified the rupture site of the right lingual artery **(E)** *(yellow arrow*) with only minor contrast extravasation, probably contained by the previously placed oral packing. After embolization, the final injection **(F)** in the ECA demonstrated opacification limited to the ostium of the right lingual artery (*blue arrow*) without any distal filling, confirming complete vessel occlusion. The glue cast is also visible (*blue arrowheads*)

A 64-year-old man with a history of laryngeal squamous cell carcinoma, previously treated with total laryngectomy, developed a locoregional recurrence while receiving palliative immunotherapy. He presented to the emergency department with sudden, profuse bleeding from the oral and nasal cavities. On arrival, he was hypotensive (blood pressure 66/44 mmHg), tachycardic, and anaemic (haemoglobin 9 g/dL). Flexible nasopharyngoscopy revealed extensive clots filling the neopharynx and an ulcerated, infiltrative lesion extending from the right tonsillar fossa to the base of tongue. Topical aminocaproic acid and oropharyngeal packing were applied for temporary hemostasis. CE-CT and CTA showed post-surgical changes and irregular thickening and enhancement of the neopharyngeal walls extending to the right tongue base, consistent with recurrent tumour and ulceration (Fig. 2A, B). Within this region, a pseudoaneurysm arising from the main trunk of the right lingual artery was identified (Fig. 2C, D). Given the life-threatening bleeding, the patient underwent an emergent DSA for EVT. The procedure was performed under sedation with a right transradial access. A guiding sheath Rist 079 (*Medtronic*) and a Vertebral 5 F catheter (*Medtronic*) were used for the pre-treatment evaluation and the right ECA catheterization. A superselective microcatheterization of the right main trunk of the lingual artery using an Excelsior SL-10 (*Stryker*) and a Synchro 0.0014 soft guidewire (*Stryker*) revealed the active bleeding site (Fig. 2E), showing minimal contrast extravasation, likely contained by previous oropharyngeal packing. Embolization with diluted NBCA glue (*Glubran2*) injection was performed, successfully excluding the affected segment of the lingual artery. Final DSA runs confirmed complete vessel occlusion with no residual distal filling (Fig. 2F). Following the procedure, the patient stabilized hemodynamically and remained free of further haemorrhagic episodes until latest follow-up.

### Limitations

This study is limited by the rarity of PLAs and the consequent reliance on case reports and small case series, resulting in heterogeneous and retrospective data that are prone to publication bias. Outcome and recurrence reporting across the literature was highly inconsistent, with limited standardized information on angiographic results, functional outcomes, retreatment rates, recurrence, and follow-up duration. In many cases, angiographic endpoints and validated clinical scales were not reported, precluding a more detailed or quantitative analysis of treatment efficacy and durability. In addition, longitudinal imaging follow-up was often inexistent. Surgical cases were included for comparative discussion but were not systematically sought as a separate category, limiting direct comparison between treatment modalities. Finally, our institutional experience is limited to two cases, which, although illustrative, does not allow broad generalization.

## Conclusions

Early recognition and appropriate management of PLAs is essential to prevent rupture and haemorrhagic shock. EVT appears to be a feasible and effective option for most PLAs. Careful assessment of vascular anatomy and collateral circulation plays a central role in treatment planning and in determining the feasibility and safety of PVO. Surgical management remains an important alternative in selected cases where endovascular access is not feasible, contraindicated, or unsuccessful.

## Supplementary Information

Below is the link to the electronic supplementary material.

Supplementary Material 1 (PNG 100 KB)

## Data Availability

All data supporting the findings and analysis of this study are available within the paper , along with the original references across the study.

## Abbreviations

CE-CT: Contrast-enhanced Computed Tomography
CTA: Computed Tomography Angiography
DSA: Digital Subtraction Angiography
ECA: External Carotid Artery
EVT: Endovascular Treatment
FMD: Fibromuscular Dysplasia
MR: Magnetic Resonance
MRA: Magnetic Resonance Angiography
NBCA: n-butyl-2-cyanoacrylate
OSAS: Obstructive Sleep Apnea Syndrome
PLA: Pseudoaneurysms of the Lingual Artery
PVO: Parent Vessel Occlusion
TCRF: Temperature-controlled Radiofrequency Reduction
TORS: Transoral Robotic Surgery
US: Ultrasound

## References

[1] Faggioli GL, Freyrie A, Stella A et al (1996) Extracranial internal carotid artery aneurysms: results of a surgical series with long-term follow-up. J Vasc Surg 23:587–594. 10.1016/s0741-5214(96)80037-1 8627893 10.1016/s0741-5214(96)80037-1

[2] Moreau P, Albat B, Thévenet A (1994) Surgical treatment of extracranial internal carotid artery aneurysm. Ann Vasc Surg 8:409–416. 10.1007/BF02133059 7811577 10.1007/BF02133059

[3] McCollum CH, American WWT (1979) Aneurysms of the extracranial carotid artery. americanjournalofsurgerycom

[4] van Sambeek MR, Segeren CM, van Dijk LC et al (2000) Endovascular repair of an extracranial internal carotid artery aneurysm complicated by heparin-induced thrombocytopenia and thrombosis. J Endovasc Ther 7:353–358. 10.1177/152660280000700502 11032253 10.1177/152660280000700502

[5] O’Neill J, Shaw-Dunn J, Robertson S et al (2016) Arterial anastomosis in the tongue. J Oral Maxillofac Surg 74:1084–1090. 10.1016/j.joms.2015.12.011 26836294 10.1016/j.joms.2015.12.011

[6] DiStefano JF, Maimon W, surgery MMJOO et al False aneurysm of the lingual artery. europepmcorg 269937

[7] Walshe P, Ramos E, Low C, Thomas L, McWilliams R, Hone S (2005) An unusual complication of tonsillectomy. Surgeon 3(4):296–298. 10.1016/S1479-666X(05)80096-4 16121779 10.1016/s1479-666x(05)80096-4

[8] Hassan F et al (2019) Endovascular embolization of Post-tonsillectomy pseudoaneurysm: A Single-Center case series. Cardiovasc Interventional Radiol Vol 42(4):528–533. 10.1007/s00270-018-2131-9 10.1007/s00270-018-2131-9 30519726

[9] Schroth G et al (1991) Aneurysm of the lingual artery. Endovascular Treat Neuroradiol Vol 33:451–452. 10.1007/BF00598625 10.1007/BF00598625 1749481

[10] Cheng L, Kennedy H, Wong K, Tahim A, Gillan G (2017) Pseudoaneurysm of the lingual artery — a case series. Int J Oral Maxillofac Surg 46:282. 10.1016/j.ijom.2017.02.950

[11] Rathod R et al (2021) Early presentation of traumatic pseudoaneurysm of deep lingual artery as a massive oral bleed. BMJ case reports 14,4 e240928. 28 Apr. 10.1136/bcr-2020-240928 10.1136/bcr-2020-240928 PMC8094351 33910799

[12] Fox CJ, Gillespie DL, Weber MA et al (2006) Delayed evaluation of combat-related penetrating neck trauma. J Vasc Surg 44(1):86–93. 10.1016/j.jvs.2006.02.058 16828429 10.1016/j.jvs.2006.02.058

[13] Gratacap M, Couloigner V, Boulouis G et al (2015) Embolization in the management of recurrent secondary post-tonsillectomy haemorrhage in children. Eur Radiol 25(1):239–245. 10.1007/s00330-014-3387-3 25163899 10.1007/s00330-014-3387-3

[14] Lepore ML (1976) Upper airway obstruction induced by warfarin sodium. Arch Otolaryngol 102(8):505–506. 10.1001/archotol.1976.00780130099015 942329 10.1001/archotol.1976.00780130099015

[15] Alamoudi U, Alsallumi Y, Rigby MH, Taylor SM, Trites JRB, Hart RD (2017) Spontaneous submental hematoma, a pseudo-Ludwig’s phenomenon in 101-year-old patient: case report and literature review. Int J Surg Case Rep 36:98–102. 10.1016/j.ijscr.2017.05.009 28551485 10.1016/j.ijscr.2017.05.009 PMC5447390

[16] Cohen AF, Warman SP (1989) Upper airway obstruction secondary to Warfarin-Induced Sublingual hematoma. Arch Otolaryngol Head Neck Surg 115(6):718–720. 10.1001/archotol.1989.01860300072020 2655669 10.1001/archotol.1989.01860300072020

[17] Gomori JM, Dermer R, Shifrin E (1983) Aneurysm of the lingual artery. Neuroradiology 25(2):111–112. 10.1007/BF00333302 6877588 10.1007/BF00333302

[18] Orron DE, Greenberg JJ, Kim D, Skillman JJ (1988) Pseudoaneurysm of the lingual artery. Comput Med Imaging Graph 12(6):349–352. 10.1016/0895-6111(88)90075-4 3061634 10.1016/0895-6111(88)90075-4

[19] Maurer J et al Aneurysma der A. lingualis als Ursache wiederholter Spätblutungen nach Tonsillektomie [Aneurysms of the lingual artery as a cause of recurrent late hemorrhage following tonsillectomy]. Laryngo- rhino- otologie vol. 68,5 (1989): 301-3. d 10.1055/s-2007-998339 2742653

[20] Adib A et al (1993) Bilateral idiopathic aneurysms of the lingual arteries. Otolaryngology–head and neck surgery : official journal of American academy of Otolaryngology-Head and neck surgery. 108(1):87–90. 10.1177/019459989310800113 10.1177/019459989310800113 8437880

[21] Salgarelli A et al (1997) Pseudoaneurysm of the lingual artery: a case report. J Oral Maxillofacial Surg : Official J Am Association Oral Maxillofacial Surg Vol 55:860–864. 10.1016/s0278-2391(97)9035 10.1016/s0278-2391(97)90351-7 9251618

[22] Mitchell RB, Pereira KD, Lazar RH, Long TE, Fournier NF (1997) Pseudoaneurysm of the right lingual artery: an unusual cause of severe hemorrhage during tonsillectomy. Ear Nose Throat J 76(8):575–576. 10.1177/014556139707600815 9282466

[23] Amirjamshidi A, Abbassioun K, Rahmat H (2000) Traumatic aneurysms and arteriovenous fistulas of the extracranial vessels in war injuries. Surg Neurol 53(2):136–145. 10.1016/S0090-3019(99)00181-0 10713191 10.1016/s0090-3019(99)00181-0

[24] Simoni P, Bello JA, Kent B (2001) Pseudoaneurysm of the lingual artery secondary to tonsillectomy treated with selective embolization. Int J Pediatr Otorhinolaryngol 59(2):125–128. 10.1016/s0165-5876(01)00478-5 11378188 10.1016/s0165-5876(01)00478-5

[25] Roelke LH, Perez J, Vascular FTJ et al Tratamento endovascular de Um Caso Raro de pseudo-aneurisma de carótida externa após Amigdalectomia. researchgatenet

[26] Herzog M, Schmidt A, Metz T et al (2006) Pseudoaneurysm of the lingual artery after temperature-controlled radiofrequency tongue base reduction: A severe complication. Laryngoscope 116(4):665–667. 10.1097/01.mlg.0000200795.12919.6a 16585878 10.1097/01.mlg.0000200795.12919.6a

[27] Li S-H et al (2007) Pseudoaneurysm of the external carotid artery branch following radiotherapy for nasopharyngeal carcinoma. Japanese J Clin Oncol Vol 37(4):310–313. 10.1093/jjco/hym019 10.1093/jjco/hym019 17553820

[28] van Cruijsen N et al Severe delayed posttonsillectomy haemorrhage due to a pseudoaneurysm of the lingual artery. European archives of oto-rhino-laryngology : official journal of the European federation of Oto-Rhino-Laryngological societies (EUFOS). 10.1007/s00405-007-0391-0 10.1007/s00405-007-0391-0 PMC2099165 17639439

[29] Matsumoto T et al (2007) Transcatheter arterial embolisation of a ruptured pseudoaneurysm of the lingual artery with n-butyl cyanoacrylate. Br J Radiol 80(950):e54–e57. 10.1259/bjr/61848822 17495057 10.1259/bjr/61848822

[30] Handa KK, Shunyu NB (2008) Post traumatic psuedoaneurysm of the lingual artery. Indian J Otolaryngol Head Neck Surg 60(4):356–359. 10.1007/s12070-008-0115-9 23120581 10.1007/s12070-008-0115-9 PMC3476801

[31] Windfuhr JP et al (2010) Post-tonsillectomy pseudoaneurysm: an underestimated entity? J Laryngology Otology Vol 124(1):59–66. 10.1017/S0022215109990922 10.1017/S0022215109990922 19765325

[32] Brindle RS, Fernandez PM, Sattenberg RJ, Flynn MB, Heidenreich JO (2010) Idiopathic lingual artery aneurysm: CT findings and endovascular therapy: A case report: A case report. Interventional Neuroradiol 16(1):103–106. 10.1177/159101991001600115 10.1177/159101991001600115 PMC3277968 20377988

[33] Murono S et al (2011) Pseudoaneurysm of the lingual artery after concurrent intra-arterial chemotherapy with radiotherapy for advanced tongue cancer. Head Neck Vol 33:1230–1232. 10.1002/hed.21372 10.1002/hed.21372 20310040

[34] Kaschner M, Strunk H Thrombinembolisation eines aneurysma spurium der arteria lingualis Nach Stumpfem halstrauma [Thrombin embolization of a pseudoaneurysm of the arterial lingualis after blunt neck trauma]. RoFo : fortschritte auf dem gebiete der. 10.1055/s-0031-1273467 10.1055/s-0031-1273467 21935863

[35] Atmaca S, Belet U, Baris S (2012) Post-tonsillectomy pseudoaneurysm of the linguofacial trunk: an ENT surgeon’s nightmare. Int J Pediatr Otorhinolaryngol Extra 7(1):12–14. 10.1016/j.pedex.2011.07.006

[36] Fassnacht W, Hammer F, Gardiner Q, desuter G (2012) Delayed endovascular coil extrusion after embolisation for post-tonsillectomy haemorrhage: case report and literature review. J Laryngol Otol 127:1–4. 10.1017/S0022215112002599 10.1017/S0022215112002599 23199626

[37] Badloo K et al (2012) Mycotic pseudoaneurysm of the lingual artery: a rare complication of parapharyngeal abscess. J Paediatrics Child Health Vol 48:1045–1046. 10.1111/j.1440-1754.2012.02599.x 10.1111/j.1440-1754.2012.02599.x 23126396

[38] Masella PC et al (2014) Posttraumatic lingual artery pseudoaneurysm treated with ultrasound-guided percutaneous thrombin injection. Annals Vascular Surg Vol 28:1317e11–1317e15. 10.1016/j.avsg.2013.10.013 10.1016/j.avsg.2013.10.013 24365080

[39] Manzato L, Trivelato FP, Alvarenga AYH, Rezende MT, Ulhôa AC (2013) Endovascular treatment of a linguofacial trunk pseudoaneurysm after tonsillectomy. Braz J Otorhinolaryngol 79(4):524. 10.5935/1808-8694.20130094 23929158 10.5935/1808-8694.20130094 PMC9442336

[40] Iwai T, Izumi T, Hayashi Y et al (2017) Bilateral idiopathic aneurysms of the lingual artery identified by three-dimensional computed tomography angiography. Oral Radiol 33(3):227–230. 10.1007/s11282-016-0255-7

[41] Margallo L, de Zárate EO, Franco M et al (2018) Lingual artery pseudoaneurysm after severe facial trauma. Craniomaxillofac Trauma Reconstr 11(3):219–223. 10.1055/s-0037-1603454 30087752 10.1055/s-0037-1603454 PMC6078696

[42] Hassan F, Younes A, Rifai M (2019) Endovascular embolization of Post-tonsillectomy pseudoaneurysm: A Single-Center case series. Cardiovasc Intervent Radiol 42(4):528–533. 10.1007/s00270-018-2131-9 30519726 10.1007/s00270-018-2131-9

[43] Ramírez-Ramírez MM, Clemente-Gutiérrez UE, Silva-González M, Zúñiga-Zamora HM, Sánchez-Conejo AR (2019) Congenital lingual artery aneurysm as an unusual cause of upper Gastrointestinal bleeding. Rev Gastroenterol Mex 84(2):248–250. 10.1016/j.rgmx.2018.02.006 10.1016/j.rgmx.2018.02.006 29678363

[44] Leedman SR, Esmaili A, Singh T, Wee D (2019) Large volume haemorrhage following transoral robotic surgery (TORS) as a result of fibromuscular dysplasia: first reported case. BMJ Case Rep 12(11):10–12. 10.1136/bcr-2019-232022 10.1136/bcr-2019-232022 PMC6887428 31767611

[45] Espallargas I, Marsico S, Zuccarino F et al (2020) Pseudoaneurysm of the lingual artery as an unusual radiological finding of oropharyngeal carcinoma. BJR|case Rep 6(4):20200063. 10.1259/bjrcr.20200063 33299595 10.1259/bjrcr.20200063 PMC7709064

[46] Rathod R, Choudhary N, Hosur B, Bansal S (2021) Early presentation of traumatic pseudoaneurysm of deep lingual artery as a massive oral bleed. BMJ Case Rep 14(4):3–6. 10.1136/bcr-2020-240928 10.1136/bcr-2020-240928 PMC8094351 33910799

[47] Ali I, Naseer H, Altaf R, Mansoor MA, Khalid D (2022) Pseudo-Aneurysm of lingual artery a rare complication of squamous cell carcinoma of tongue and a cause of oral bleed. J Med Res Surg 3(1):13–14. 10.52916/jmrs224069

[48] Yamamoto N, Yamaguchi S, Nishikawa M, Ichimura N, Ohara G, Hibi H (2022) Aneurysm of the lingual artery in a patient with fibromuscular dysplasia: A case report. J Oral Maxillofac Surg Med Pathol 34(1):45–48. 10.1016/j.ajoms.2021.06.005

[49] Singh SP, Khurana R, Pandey NN, Singh Malhi A, Ramakrishnan P, Kumar S (2022) Lingual artery pseudoaneurysm following blunt neck trauma. J Endovasc Ther 29(5):692–693. 10.1177/15266028211061274 34836483 10.1177/15266028211061274

[50] Maleux O, Hermans R, Vander Poorten V, Maleux G (2022) Glue embolization of a bleeding lingual artery pseudoaneurysm related to tongue surgery. Acta Chir Belg 122(2):133–135. 10.1080/00015458.2020.1765675 32375568 10.1080/00015458.2020.1765675

[51] Bravo J, Hirshman B, Wali A, Khalessi A, Pannell J (2022) E-279 ultrasound guided direct access to embolize lingual artery pseudoaneurysm for oropharyngeal bleeding. 14(Suppl 1):A230–A231. 10.1136/neurintsurg-2022-snis.390

[52] Ahmed F, Koneru M, Garg R, Shaikh H (2023) Imaging characteristics of tongue hematoma and pseudoaneurysm following tooth extraction requiring emergency liquid embolization. Cureus 15(5). 10.7759/cureus.39731 10.7759/cureus.39731 PMC10310349 37398751

[53] SANCHEZ TRINIDAD J, GONZALEZ A, BUSMAIL HAYLOCK A et al (2024) Hemorrhagic shock due to lingual artery aneurysm. Chest 166(4):A2173–A2174. 10.1016/j.chest.2024.06.1340

[54] Daou AM, Hosri J, Mourad M Spontaneous oropharyngeal bleeding caused by lingual artery Pseudo-Aneurysm. Ear Nose Throat J Published Online 2024:0–3. 10.1177/01455613231226045 10.1177/01455613231226045 38321707

[55] Buxo Z, Rexroth J, Johnson B, Fessler R, Carron M (2024) Pseudoaneurysm of the lingual artery in a patient with facial trauma from gunshot wounds. J Craniofac Surg 36. 10.1097/SCS.0000000000010788 10.1097/SCS.0000000000010788 39730123

[56] Yang X, Zhao J, Quan R, Liu Q (2025) Endovascular embolization of post-tonsillectomy pseudoaneurysm in adults. CVIR Endovasc 8(1). 10.1186/s42155-025-00539-w 10.1186/s42155-025-00539-w PMC11961778 40167876

[57] Bhali H, El, Kaibech M, Bourfoune Z, Kourriche W, Mouhanie S, Azghari A (2025) Cervicofacial cellulitis complicated by a false lingual artery aneurysm: A very rare complication. Radiol Case Rep 20(4):2231–2234. 10.1016/j.radcr.2025.01.053 39996121 10.1016/j.radcr.2025.01.053 PMC11847658

[58] Page MJ et al (2021) The PRISMA 2020 statement: an updated guideline for reporting systematic reviews. BMJ. 29 Mar 372. (Clinical research ed.)10.1136/bmj.n71 10.1136/bmj.n71 PMC8005924 33782057

[59] Kennedy H, Cheng L, Tahim A, Wong K, Gillan G. Pseudoaneurysm of the lingual artery– a case series. *Br J Oral Maxillofac Surg*. 2017;55(10):e102. doi:10.1016/j.bjoms.2017.08.054

[60] Wilcox WR (2003) Connective tissue and its heritable disorders: Molecular, Genetic, and medical aspects. Am J Hum Genet Vol 72(2):503–504

[61] Fankhauser GT et al (2015) Surgical and medical management of extracranial carotid artery aneurysms. J Vascular Surg Vol 61(2):389–393. 10.1016/j.jvs.2014.07.092 10.1016/j.jvs.2014.07.092 25151599

[62] Gagné-Loranger M et al (2016) Should endovascular therapy be considered for patients with connective tissue disorder? Can J Cardiol Vol 32(1):1–3. 10.1016/j.cjca.2015.06.026 10.1016/j.cjca.2015.06.026 26577892

[63] Cohen JoséE et al (2012) Endovascular management of postoperative pseudoaneurysms of the external carotid artery. J Clin Neurosci : Official J Neurosurgical Soc Australasia Vol 19(5):649–654. 10.1016/j.jocn.20 10.1016/j.jocn.2011.11.007 22502912

[64] Katsumi Y, Tanaka R, Hayashi T et al (2011) Variation in arterial supply to the floor of the mouth and assessment of relative hemorrhage risk in implant surgery. Clin Oral Implants Res 24:434–440. 10.1111/j.1600-0501.2011.02348.x 22092873 10.1111/j.1600-0501.2011.02348.x

