# Supplementary figures and images for: Pseudoaneurysms of the lingual artery: two case reports and a systematic review
Source: Neuroradiology. 2026 Mar 6;68(4):1077–92. doi: 10.1007/s00234-026-03932-x (PMC13139205; doi:10.1007/s00234-026-03932-x)

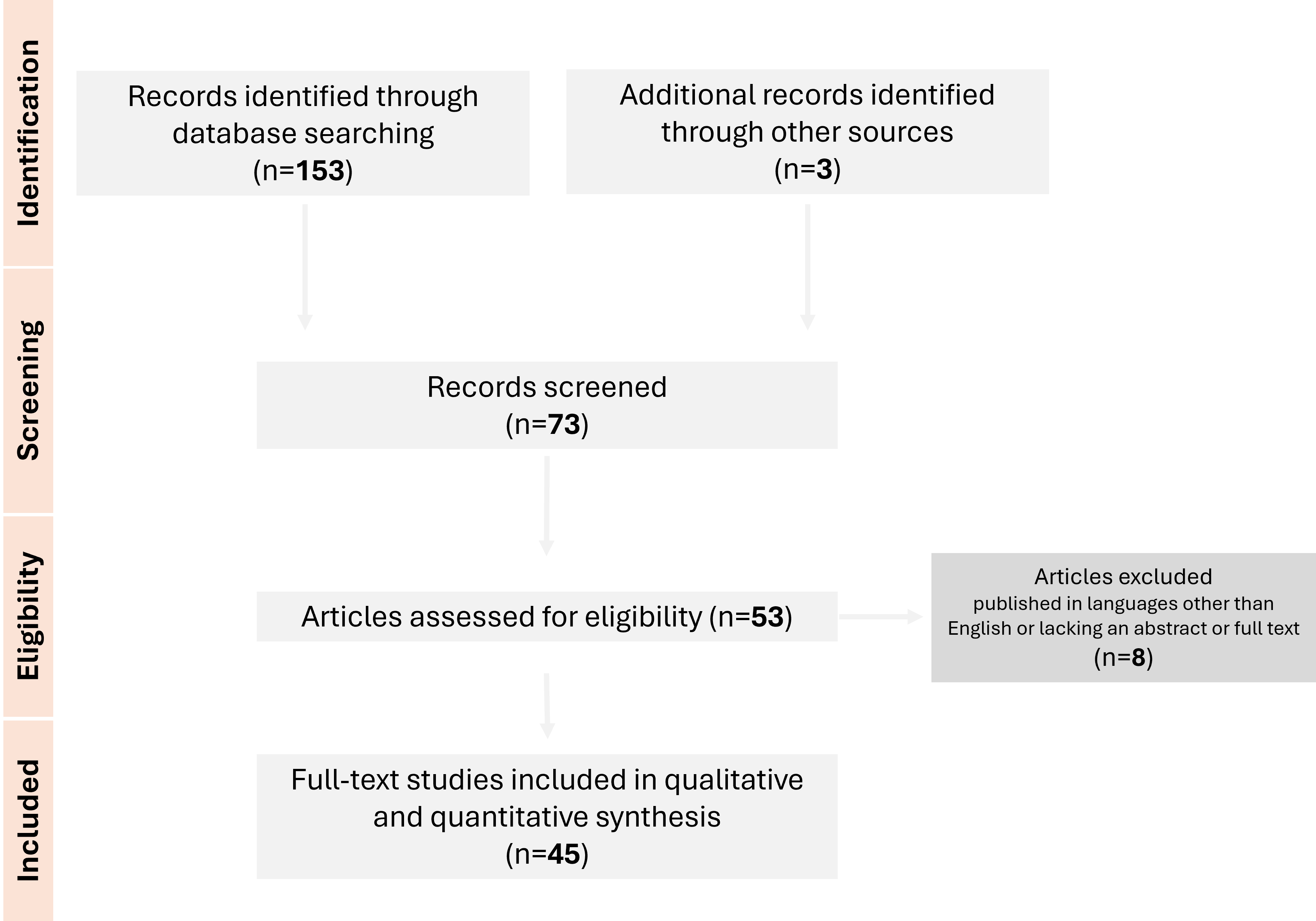

Supplement: Supplementary file 1 — Supplementary Material 1 (PNG 100 KB) [file 234_2026_3932_MOESM1_ESM.png]
